# Extracorporeal membrane oxygenation for COVID-19-related acute respiratory distress syndrome: a narrative review

**DOI:** 10.1186/s40560-023-00654-7

**Published:** 2023-02-08

**Authors:** Francesco Alessandri, Matteo Di Nardo, Kollengode Ramanathan, Daniel Brodie, Graeme MacLaren

**Affiliations:** 1 Department of General and Specialistic Surgery, Sapienza University of Rome, Rome, Italy; 2 Pediatric Intensive Care Unit, Children’s Hospital Bambino Gesù, IRCCS, Rome, Italy; 3 Cardiothoracic Intensive Care Unit, National University Hospital, Singapore, Singapore; 4 Columbia University College of Physicians and Surgeons/New York-Presbyterian Hospital, New York, NY USA; 5 Center for Acute Respiratory Failure, Columbia University Medical Center, New York, NY USA

**Keywords:** Extracorporeal membrane oxygenation, ECMO, COVID-19, ARDS, Respiratory failure, Extracorporeal life support

## Abstract

A growing body of evidence supports the use of extracorporeal membrane oxygenation (ECMO) for severe acute respiratory distress syndrome (ARDS) refractory to maximal medical therapy. ARDS may develop in a proportion of patients hospitalized for coronavirus disease 2019 (COVID-19) and ECMO may be used to manage patients refractory to maximal medical therapy to mitigate the risk of ventilator-induced lung injury and provide lung rest while awaiting recovery. The mortality of COVID-19-related ARDS was variously reassessed during the pandemic. Veno-venous (VV) ECMO was the default choice to manage refractory respiratory failure; however, with concomitant severe right ventricular dysfunction, venoarterial (VA) ECMO or mechanical right ventricular assist devices with extracorporeal gas exchange (Oxy-RVAD) were also considered. ECMO has also been used to manage special populations such as pregnant women, pediatric patients affected by severe forms of COVID-19, and, in cases with persistent and seemingly irreversible respiratory failure, as a bridge to successful lung transplantation. In this narrative review, we outline and summarize the most recent evidence that has emerged on ECMO use in different patient populations with COVID-19-related ARDS.

**Supplementary Information:**

The online version contains supplementary material available at 10.1186/s40560-023-00654-7.

## COVID-19-related ARDS

The clinical presentation of coronavirus disease 2019 (COVID-19) is notably heterogeneous, ranging from no symptoms to potentially fatal acute respiratory distress syndrome (ARDS) and, in a small minority of cases, from myocardial inflammation (e.g., myocarditis) to cardiogenic shock [1, 2]. ARDS is characterized by an increase in the anatomical shunt (e.g., increase in noncardiogenic pulmonary edema) and by a reduction of functioning lung size (i.e., “baby lung”), which accounts for high respiratory system elastance. In these patients, increasing lung size by recruiting or maintaining the patency of previously collapsed lung units is often achieved using moderate-to-high levels of positive end-expiratory pressure (PEEP), prone positioning, and occasionally via lung recruitment maneuvers [3]. In patients with early COVID-19-related ARDS, hypoxemia could be explained by an increase in the physiologic shunt due to loss of hypoxic pulmonary vasoconstriction and by an increase in dead space fraction due to development of pulmonary vascular microthrombi [4, 5]. Of note, preliminary hyper-inflammatory and hypo-inflammatory sub-phenotypes, defined by unique clinical features and biomarkers, have been described in COVID-19-related ARDS. The hyper-inflammatory sub-phenotype has higher inflammatory and lactate markers than the hypo-inflammatory sub-phenotype and is associated with significantly higher 90-day mortality than the hypo-inflammatory sub-phenotype (75% vs. 48%) [6]. The two sub-phenotypes may respond differently to corticosteroid treatment, with the suggestion of an improved survival in the hyper-inflammatory sub-phenotype, but not in the hypo-inflammatory sub-phenotype [6, 7].

Regarding respiratory mechanics, recently Reddy et al. found no evidence of distinct clinical phenotypes [8]. In a well-conducted systematic review and meta-analysis of 37 studies of COVID-19-related ARDS published between 2019 and 2022, the mean compliance of the respiratory system (C_RS_) was inversely proportional to the severity of ARDS (39.3 mL/cm H_2_O [36.6–42.0] in mild ARDS, 34.9 mL/cm H_2_O [32.8–36.9] in moderate ARDS, and 27.3 mL/cm H_2_O [23.3–31.2] in severe ARDS). Therefore, the mean C_RS_ measured close to the time of the initiation of invasive mechanical ventilation was normally distributed in these patients just like in conventional ARDS, with no evidence of distinct clinical phenotypes based on respiratory mechanics. Based on these findings, traditional lung protective ventilation strategies tailored to the patient’s lung mechanics are recommended in patients with severe COVID-19 ARDS. [8, 9]. However, COVID‐19 patients seem to have a higher extravascular lung water index (EVLWi) and pulmonary vascular permeability index (PVPI) values than non-COVID-19 patients, from the beginning of the disease [10].

### Extracorporeal membrane oxygenation (ECMO)

ECMO is currently used to manage severe respiratory and/or cardiac failure unresponsive to optimal conventional management. In general, veno-venous (VV) ECMO is used to manage severe respiratory failure, while venoarterial (VA) ECMO is used to manage severe cardiac failure [11]. In VV ECMO, deoxygenated blood is drained from a central vein and is pumped through a membrane lung in which gas exchange occurs. Oxygenated blood is then reinfused back into the venous system [11, 12]. Conversely, in VA ECMO, blood is reinfused directly back into the arterial system, augmenting the cardiac output provided by the native heart [11]. In general, patients with COVID-19-related ARDS refractory to medical therapy have been supported with VV ECMO, while patients presenting with both respiratory failure and right ventricular dysfunction (or COVID-19 myocarditis) have potential required support with VA ECMO or a combination of both [13, 14].

### Evidence for ECMO in the management of ARDS

The use of ECMO to manage patients with severe ARDS has increased in recent years, in part due to the experience with patients supported by ECMO during the 2009 influenza A(H1N1) pandemic [15, 16]. Despite several methodological limits, the randomized controlled trial and parallel economic evaluation of Conventional ventilatory support versus Extracorporeal membrane oxygenation for Severe Adult Respiratory failure (CESAR) trial provided evidence of likely benefit from the use of ECMO in patients with acute hypoxemic respiratory failure [16]. Therefore, the Extracorporeal Membrane Oxygenation for Severe Acute Respiratory Distress Syndrome (EOLIA) trial was designed to better define the role of ECMO in the management of severe ARDS [17]. In this study, patients were randomized to ECMO or standard care (protocolized mechanical ventilation). Although this trial was stopped for futility (despite reporting a non-significant 11% absolute difference in 60-day mortality), a post hoc analysis using a Bayesian approach suggested a survival benefit with the use of ECMO [17, 18]. Supported by the results of these studies and subsequent meta-analyses, VV ECMO has achieved a role in the management of patients with severe ARDS when the ratio of arterial oxygen partial pressure to fractional inspired oxygen (PaO_2_/FIO_2_ ratio) is lower than 50 mmHg for more than three hours, the PaO_2_/FIO_2_ is lower than 80 mmHg for more than six hours and the pH is lower than 7.25 with a partial pressure of carbon dioxide (PaCO_2_) greater than or equal to 60 mmHg for more than six hours [17, 19] (Table 1).

**Table 1:** Indications and contraindications for VV ECMO

*Indications*
Endotracheal intubation and mechanical ventilation < 7 days
Hypoxic respiratory failure due to any cause when the predicted mortality risk is > 80%: - PaO_2_/FiO_2_ ≤ 50 mmHg for > 3 h - PaO_2_/FiO_2_ ≤ 80 mmHg for > 6 h - Arterial blood pH of < 7.25 with a PaCO_2_ ≥ 60 mmHg for > 6 h, with the RR increased to 35 bpm) [Mechanical ventilation settings adjusted to keep a P_plat_ ≤ 32 cmH_2_O despite ventilator optimization (FiO_2_ ≥ 0.80, a VT 6 ml/Kg of PBW and a PEEP ≥ 10 cmH_2_O)]
Severe air leak syndrome
Need for intubation in patients on lung transplant list
*Absolute contraindications*
Irreversible lung disease
*Relative contraindications*
Age ≥ 65–70 y
Immunocompromised status
No legal medical decision-maker available
Advanced chronic systolic heart failure
Clinical Frailty Scale Category ≥ 3
Significant comorbidities
- CKD ≥ III
- Cirrhosis
- Dementia
- Baseline neurologic disease precluding rehabilitation potential
- Uncontrolled diabetes with chronic end-organ dysfunction
- Severe deconditioning
- Protein-energy malnutrition
- Severe peripheral vascular disease
- Other life-limiting medical illness
- Non-ambulatory status
Severe multiple organ failure
Severe acute neurologic injury, e.g., anoxic, stroke
Uncontrolled bleeding or contraindication to anticoagulation
Inability to accept blood products
Ongoing CPR

MV: mechanical ventilation; PaO_2_/FiO_2_: ratio of arterial oxygen partial pressure to fractional inspired oxygen; PaCO_2_: arterial carbon dioxide partial pressure; RR: respiratory rate; bpm: breaths per minute; P_plat_: plateau pressure: VT: tidal volume; PBW: predicted body weight; PEEP: positive end-expiratory pressure; CKD = chronic kidney disease; CPR: cardio pulmonary resuscitation

### VV ECMO and COVID-19-related ARDS

Despite dismal outcomes reported from preliminary reports on the use of ECMO [20, 21], data collected by international registries and larger cohort studies highlighted that outcomes for patients with COVID-19-related ARDS supported with ECMO were similar to those reported in patients with non-COVID-19-related ARDS [22]. Further data also highlighted that both ECMO duration and mortality were unexpectedly increasing over time [23–29]. At the beginning of the pandemic (“first wave”), several studies have reported a 90-day mortality rate between 36 and 47% [23–26].

From mid-2020, studies reported higher mortality with the use of ECMO for COVID-19, raising concerns about patient selection [25–32]. This apparent worsening of mortality could be explained by several factors: the potential self-induced lung injury caused by prolonged use of non-invasive respiratory support before endotracheal intubation, more liberal ECMO use, the increase in bacterial superinfections due to more frequent use of COVID-19 immunosuppressive treatments over time, and the use of ECMO in less experienced centers [27]. Of note, the use of ECMO for COVID-19-related ARDS was associated with a high number of thrombotic complications in the extracorporeal circuit compared to non-COVID-19 related ARDS [33, 34]. In general management of anticoagulation is complex during ECMO and requires special attention in COVID-19 patients who have a greater risk of thrombosis than other etiologies [35]. Based on these data, many centers have tried to increase their anticoagulation targets and have also tried alternative drugs (e.g., bivalirudin), however, the bleeding risk remained a concern [36]. So far, there are insufficient data to suggest deviation from usual anticoagulation practices for patients with COVID-19 receiving ECMO [37].

Propensity score matching analysis [38] and recent systematic reviews and meta-analyses have supported the positive results of these cohort studies conducted at the beginning of pandemic; however, since the causes of temporal increase in mortality observed in the later phase of pandemic were not addressed in these meta-analyses, the effectiveness of ECMO for COVID-19 remained controversial [39, 40].

Using observational data to emulate a randomized controlled trial represents an established statistical approach to estimate treatment effectiveness of an intervention in an uncontrolled setting when randomized controlled trials cannot be performed because of restrictive inclusion criteria, likelihood of crossover among treatments, costs, slow enrollment rate, lack of equipoise, and ethical issues [41, 42]. Investigating the effectiveness of ECMO during a global pandemic has been challenging and emulation trials have been used for this purpose. These new statistical analyses represent an attractive alternative to randomized trials to answer the question of interest, using available observational data.

Three target emulation trials (Table 2) have been recently developed to evaluate the efficacy of ECMO versus conventional mechanical ventilation in patients with severe COVID-19-related ARDS [43–45]. Shaefi et al., including patients with severe hypoxemia (PaO_2_/FIO_2_ < 80 mmHg), observed a reduction in 60-day hospital mortality associated with ECMO (hazard ratio 0.55; 95% confidence interval (CI) 0.40–0.77) [43]. Hajage et al. observed that patients with severe hypoxemia (PaO_2_/FIO_2_ < 80 mmHg) receiving ECMO had a higher survival probability on day 7 compared to the alternative strategy without ECMO (87% vs 83%, risk difference, 4%; 95% CI, 0–9%), but worsened at day 90 (63% on ECMO versus 65% on conventional arm, risk difference: − 2%; 95% CI, − 10 to 5%) [44]. Of note, this apparent reversal of the efficacy of ECMO at 90 days was no longer present when the analysis was performed including only high-volume centers. The authors concluded that VV ECMO should be started early (within the first 4 days of invasive mechanical ventilation) and only in severe hypoxemic patients (PaO_2_/FIO_2_ < 65) [44]. In this cohort, ECMO was associated with higher survival when performed in high-volume ECMO centers or in regions where specific inter-institutional ECMO networks were set up to handle high demand. This suggests that centralization of regional ECMO services to high-volume centers may represent the optimal approach. A third and larger emulated target trial from the COVID-19 Critical Care Consortium Investigators [45], including 7345 COVID-19 patients of whom 844 patients received ECMO [39], showed that patients with severe hypoxemia (PaO_2_/FIO_2_ < 80 mmHg) supported with ECMO reported a 60-day mortality lower than that of patients managed only with mechanical ventilation (26% versus 33.2%; risk difference: − 7.1%, 95% CI: − 8.2% to − 6.1%, RR 0.78; CI: 0.75–0.82). Factors associated with favorable outcome included age < 65 years, PaO_2_/FiO_2_ < 80, duration of invasive mechanical ventilation ≤ 10 days and driving pressure > 15 cm H_2_O [45]. Contrarily, we do not have any data to suggest a specific ventilatory setting during ECMO for COVID-19 ARDS patients. Since COVID-19 ARDS has similar lung mechanic characteristics of non-COVID-19 ARDS, a recent consensus statement suggested to use “lung rest settings” (very low-pressure, low-volume ventilation, low rate ventilation and a moderate PEEP to avoid the increase of lung collapse) for COVID-19 patients receiving ECMO for ARDS [37]. In summary, evidence provided by these emulated targeted trials supported the role of ECMO for patients with severe ARDS due to COVID-19. Greater benefit was seen in patients who received ECMO earlier in their course of mechanical ventilation, those with severe hypoxemia, or in those receiving high-intensity mechanical ventilation.

**Table 2:** Studies reporting mortality for VV ECMO in COVID-19 related ARDS (Additional File 1)

	Data source	Study period	ECMO patients	Mortality
Preliminary studies				
Ruan et al. 2020	China	–	7	100%
Wu et al. 2020	China	–	1	100%
Yang et al. 2020	China	Dec 24, 2019–Jan 26, 2020	6	83.4%
Zhou et al. 2020	China	Dec 29, 2019–Jan 31, 2020	3	100%
Guan et al. 2020	China	Dec 11, 2019–Jan 29, 2020	5	–
Retrospective observational studies—Registries				
Barbaro et al. 2020	ELSO Registry	Jan 16, 2020–May 1, 2020	1035	37.4%
Lorusso et al. 2021	Euro ELSO survey	Mar 15, 2020–Sep 14, 2020	1531	45%
Schmidt et al. 2020	France	Mar 8, 2020–May 2, 2020	83	36%
Barbaro et al. 2021	ELSO Registry	A1 Early adopting centers (Before May 1, 2020) A2 Early adopting centers (May 2–Dec 31, 2020) B Late adopting centers (May 2–Dec 31, 2020)	1182 2824 803	36.9% 51.9% 58.9%
Broman et al. 2021	Euro ELSO survey	Mar 12, 2020–Sep 14, 2020 Sep 15, 2020–Mar 8, 2021	1442 1723	47% 56%
Riera et al. 2021	Spain, Portugal	Mar 1, 2020–Jun 30, 2020 Jul 1, 2020–Dec 1, 2020	151 168	41.1% 60.1%
Schmidt et al. 2021	France	Mar 8, 2020–Jun 30, 2020 Jul 1, 2020–Jan 28, 2021	88 71	36% 48%
Karagiannitis et al. 2021	Germany	Feb 2020–Dec 2020	119	71%
Lorusso et al. 2022	Euro ELSO survey	Mar 1, 2020–Sep 13, 2020	1215	50%
Ohshimo et al. 2022	Japanese National Registry	Feb, 2020–Nov 2021	1214	32–40%
Emulation studies				
Shaefi et al. 2021	United States	Mar 1, 2020–Jul 1, 2020	130	34.6%
Urner et al. 2022	COVID-19 Critical Care Consortium registry	Jan 3, 2020–Aug 29, 2021	844	26%
Hajage et al. 2022	French, Belgian, Swiss	Feb 25, 2020, and May 4, 2020	269	44%

*ELSO* Extracorporeal Life Support Organization

### VA ECMO and COVID-19-related ARDS 

ARDS was the most common clinical presentation of COVID-19 patients in critical care, and VV ECMO was the most commonly used ECMO configuration (> 95%) [26]. Among patients with COVID-19-related ARDS, heart failure occurred as a complication of various intercurrent factors: sepsis-related injury, cytokine storm, microvascular thrombosis, severe hypoxia, and direct cardiomyocyte damage [46–48]. Right ventricular (RV) dysfunction occurred in one-fifth of patients with COVID-19 related ARDS and was associated with a threefold increase in mortality [47]. Despite increasing knowledge about cardiac involvement in COVID-19, there are few data on the use of mechanical circulatory support in COVID-19 patients. Furthermore, VA-ECMO may have been underutilized during the pandemic [49]. Mortality and the rate of complications was higher in COVID-19-related ARDS patients with heart failure, especially in those supported with VA ECMO or where a change in configuration was delayed (3.1%) [50]. In ARDS patients who developed right ventricular dysfunction, a promising approach was the use of a right ventricular assist device with an oxygenator (Oxy-RVAD). Cain et al. compared 18 COVID-19 ARDS patients on Oxy-RVAD with 18 similar patients on invasive mechanical ventilation alone [51]. Patients treated with Oxy-RVAD reported a significantly lower in-hospital mortality and 30-day mortality than control patients [51].

## Special populations

### Use of ECMO in pregnant patients

ARDS is the most frequent cause of both admission to ICU in pregnant women and of life-threatening events for both the mother and fetus [52]. Pregnancy is an independent risk factor for ARDS and its incidence is between 70 and 120 cases per 100.000 deliveries [53]. Other pregnancy-specific risk factors for ARDS include preeclampsia, amniotic fluid embolism, tocolytic-associated pulmonary edema, and peripartum sepsis [54]. Several physiologic changes may increase the risk of ARDS in pregnant women, including the reduction of functional residual capacity (FRC) and the increase in plasma volume [55]. SARS-CoV-2 may further increase this risk by impairing the immune system, respiratory function, and coagulation system [56]. During the 2009 influenza A(H1N1) pandemic, ECMO was widely and successfully used as a rescue strategy for refractory ARDS and this was replicated during the COVID-19 pandemic [57]. In general, outcomes of pregnant and peripartum patients supported on ECMO are good and comparable to or better than many of the other cohorts [58]. In a retrospective cohort study, O’Neil et al. described 100 COVID-19 pregnant or peripartum patients supported by VV ECMO. ECMO-related complications, in particular renal complications, and hospital mortality were lower than in non-pregnant patients supported with VV ECMO for ARDS [59]. Based on these findings, the Society for Maternal–Fetal Medicine guidelines recommended the use of ECMO in refractory COVID-19-related ARDS in pregnant patients less than 32 weeks of gestation to facilitate fetal development in utero or after delivery [60]. Of note, pregnant women with severe COVID-19 should be referred early to an experienced ECMO center. In pregnant patients, ECMO blood flow should be kept high to maintain maternal SaO_2_ > 90% and thus preserve adequate fetal oxygenation. PaCO_2_ should be targeted between 28 and 32 mmHg to facilitate fetal CO_2_ elimination and oxygen intake. Resting ventilation (e.g., plateau pressure < 25 cmH_2_O, PEEP 10–15 cmH_2_O, FiO_2_ 30–40%, and a respiratory rate of 5–10 breaths/min) should be used during ECMO to mitigate the risk of ventilator-induced lung injury (VILI) and facilitate lung recovery [60]. Multidisciplinary discussion is essential when evaluating the timing and mode of delivery.

### ECMO in pediatric patients

Evidence from the first two years of the COVID-19 pandemic showed that children were less affected than adults and usually developed mild disease which was less likely to require hospital admission [61]. However, when children required hospital admission, up to one-quarter required pediatric intensive care unit (PICU) admission [61]. The mortality rate of pediatric COVID-19 was significantly lower compared with adults (< 1% in children); however, a higher mortality (up to 10%) was observed in low- and middle-income countries and in patients with pre-existing health problems [62]. In general, children with severe acute COVID-19 are admitted to PICU for pediatric ARDS (PARDS) or multisystem inflammatory syndrome (MISC) related to COVID-19. PARDS related to COVID-19 is not different from other etiologies of PARDS, therefore, the general principles of management and end goals of respiratory therapy are the same as other causes of PARDS [63]. Recent data suggest that patients with refractory hypoxemia or cardiogenic shock related to MIS-C may benefit from ECMO; however, precise ECMO indications remain unclear due to the limited number of patients treated so far [64, 65]. Although these studies may be affected by publication bias, ECMO survival in PARDS-related to COVID-19 was higher than that of PARDS from other etiologies. Of note, ECMO survival for PARDS-related to COVID-19 was higher and ECMO complications were lower in children than adults receiving ECMO for COVID-19 [66].

### Prolonged ECMO and lung transplantation

Few data exist on long-term pulmonary function in COVID-19-related ARDS patients receiving ECMO support [67]. Despite this, lung transplantation has been used as a potentially life-saving therapy in COVID-19 patients with persistent lung failure and inability to wean from ECMO, despite several weeks or months of support in the intensive care unit [68]. Clear evidence and guidelines on the indications, timing and patient selection for lung transplantation in COVID-19 patients with irreversible ARDS are scant. Some authors have suggested the use of lung transplantation in patients: (1) aged < 65 years, (2) with only single organ dysfunction, and (3) at least 4–6 weeks after the onset of respiratory failure. Good neurological status and ability to participate in a physical rehabilitation program are essential points for postoperative success (Table 3) [69]. Despite these suggestions, lung transplant candidacy remains controversial in COVID-19 patients receiving prolonged ECMO. Mohanka et al. compared the outcomes of 10 patients who required ECMO for less than 30 days with 10 patients who received prolonged ECMO support (> 30 days) [70]. Mohanka et al. observed that patients supported with prolonged ECMO for COVID-19-related ARDS recovered without the need of a lung transplant beyond the 6-week period and suggested a more conservative timeline when considering lung transplantation [70]. Based on these findings, a multidisciplinary approach should be used to assess whether patients with COVID-19-related ARDS receiving ECMO may have the potential for recovery or not, since healing without transplantation is obviously more beneficial [71]. Of note, in the past two pandemic years, several concerns have emerged regarding the use of lung transplantation in COVID-19 patients, such as the shortage of donors, the penalization of patients on the waiting list, the lack of follow-up data beyond one year after lung transplant and the potential discrimination in organ allocation systems, along with underestimation of the potential for lung parenchyma healing even after prolonged ECMO support [72–75]. Lastly, there may be ethical dilemmas surrounding candidacy for lung transplantation in those who have declined COVID-19 vaccination [76].

**Table 3:** Patients with COVID-19-related ARDS who are candidates for lung transplantation

The patient should fulfill standard criteria for LTx	Comment
Age > 65 years	Poor outcomes for older patients
At least 4–6 weeks to exclude native lung recovery	sufficient time should be allowed for lung recovery
Radiographic findings correlated with the patient’s clinical course	Extensive honeycombing, cystic changes, reticular opacities, and traction bronchiectasis, > 80% of lung involvement, a right atrium to left atrium ratio > 1, associated with extended period of static respiratory mechanics or ECMO parameters
Negative SARS-CoV-2 virology status	Mortality after surgical procedures is significantly higher for PCR-positive patients, even in those who are asymptomatic
Irreversible concomitant organ failure must be absent	In selected cases, multiorgan transplantation can be considered
The patient should be able to actively participate in physical rehabilitation	Patients able to at least sit out of bed and stand with assistance for them to remain viable transplant candidates
The patient should be able to provide first-person consent to LTx and transfusion	- Patients need to understand the impact of transplantation on quality of life - Immunosuppression and complications ahead can be psychologically traumatic and sometimes insurmountable
Antibodies should be carefully evaluated due to the likely history of exposure in the critical illness period leading up to listing	
The patient should have minimal acute comorbidities	
Decisions regarding these patients should be critically re-evaluated on a periodic basis	Early involvement of the lung transplant team is desirable because it permits adequate time to follow these patients longitudinally
Experience with high-risk transplantation	Referral to a few specialized centers could greatly improve outcomes for patients with COVID-19 who undergo lung transplantation
Broad donor pool and low waiting list mortality	This factor will maintain fair and equitable donor organ allocation and provide the chance for life-saving organ transplantation to patients who are more likely to survive

*LTx* lung transplantation

## Conclusions

ECMO is a well-established strategy for supporting patients with acute respiratory failure and has been successfully used in the management of COVID-19-related ARDS. Patients undergoing ECMO for COVID-19-related ARDS appear to have worse outcomes than those with non-COVID-19-related ARDS. Nonetheless, evidence provided by emulated targeted trials have highlighted a probably beneficial effect of ECMO in selected patients with severe COVID-19-related ARDS. A higher benefit was observed in patients with severe hypoxemia and those receiving high-intensity mechanical ventilation earlier in the course of illness. A greater likelihood of success was seen in high-volume, specialized centers. In patients with COVID-19-related ARDS and right heart failure, VV ECMO has been also used to improve myocardial contractility (e.g., pH normalization and PaCO_2_ reduction favoring a decrease in pulmonary vascular resistance); however, in refractory cases, VA ECMO and hybrid configurations have been successfully used. Several studies also observed a potential benefit of ECMO in pregnancy and in children. Furthermore, in prolonged respiratory failure, ECMO has also been used as a bridge to lung transplantation. Studies are needed to identify early interventions that may improve in-hospital outcomes and reduce the pressure on health systems due to health complications later.

## Supplementary Information

**Additional file 1.** References list of table 2 in order of appearance.

## Data Availability

Not applicable.

## Abbreviations

FIO_2_: High fraction of inspired oxygen
VV-ECMO: Veno-venous extracorporeal membrane oxygenation
VA-ECMO: Veno-arterial extracorporeal membrane oxygenation
COVID-19: Coronavirus infectious disease 2019
SARS-Cov2: Severe acute respiratory syndrome coronavirus 2
PaO_2_/FIO_2_ratio: Ratio of arterial oxygen partial pressure to fractional inspired oxygen
PaCO_2_: Partial pressure of carbon dioxide
SpO_2_: Peripheral oxygen saturation
OR: Odds ratio
CI: Confidence interval
