# Supplementary material for: Extracorporeal membrane oxygenation for COVID-19-related acute respiratory distress syndrome: a narrative review
Source: J Intensive Care. 2023 Feb 8;11:5. doi: 10.1186/s40560-023-00654-7 (PMC9907879; doi:10.1186/s40560-023-00654-7)
Supplement: Supplementary file 1 — Additional file 1. References list of table 2 in order of appearance. [file 40560_2023_654_MOESM1_ESM.docx]

**References list of Table 2 in order of appearance**

1. Barbaro RP, MacLaren G, Boonstra PS, Iwashyna TJ, Slutsky AS, Fan E, et al.; Extracorporeal Life Support Organization. Extracorporeal membrane oxygenation support in COVID-19: an international cohort study of the Extracorporeal Life Support Organization registry. Lancet 2020; 396:1071-8
2. Lorusso R, Combes A, Lo Coco V, De Piero ME, Belohlavek J; EuroECMO COVID-19 WorkingGroup; Euro-ELSO Steering Committee. ECMO for COVID-19 patients in Europe and Israel. Intensive Care Med. 2021;47(3):344-8.
3. Schmidt M, Hajage D, Lebreton G, Monsel A, Voiriot G, Levy D, et al. Extracorporeal membrane oxygenation for severe acute respiratory distress syndrome associated with COVID-19: a retrospective cohort study. Lancet Respir Med. 2020; 8(11):1121-31.
4. Barbaro RP, MacLaren G, Boonstra PS, Combes A, Agerstrand C, Annich G, et al.; Extracorporeal Life Support Organization. Extracorporeal membrane oxygenation for COVID-19: evolving outcomes from the international Extracorporeal Life Support Organization registry. Lancet 2021; 398:1230-8.
5. Broman LM, Eksborg S, Lo Coco V, De Piero ME, Belohlavek J, Lorusso R; et al. Extracorporeal membrane oxygenation for COVID-19 during first and second waves. Lancet Respir Med 2021; 9:e80–e81.
6. Riera J, Roncon-Albuquerque R Jr, Fuset MP, Alcantara S, Blanco-Schweizer P; ECMOVIBER Study Group. Increased mortality in patients with COVID-19 receiving extracorporeal respiratory support during the second wave of the pandemic. Intensive Care Med 2021;47:1490-
7. Schmidt M, Langouet E, Hajage D, James SA, Chommeloux J, Bréchot N, et al. Evolving outcomes of extracorporeal membrane oxygenation support for severe COVID-19 ARDS in Sorbonne hospitals, Paris. Crit Care 2021; 25(1):355.
8. Karagiannidis C, Strassmann S, Merten M, Bein T, Windisch W, Meybohm P, et al. High in-hospital mortality rate in patients with COVID-19 receiving extracorporeal membrane oxygenation in Germany: a critical analysis. Am J Respir Crit Care Med 2021;204: 991-4.
9. Lorusso R, De Piero M, Mariani M, Di Mauro M, Folliguet T, Taccone FS et al. In-hospital and 6-month outcomes in patients with COVID-19 supported with extracorporeal membrane oxygenation (EuroECMO-COVID): a multicentre, prospective observational study Lancet Respir Med 2022 Nov 16, 2022 https://doi.org/10.1016/ S2213-2600
10. Ohshimo S, Liu K, Ogura T, Iwashita Y, Kushimoto S, Shime N, et al. Trends in survival during the pandemic in patients with critical COVID-19 receiving mechanical ventilation with or without ECMO: analysis of the Japanese national registry data. Crit Care 2022;26(1):354.
11. Shaefi S, Brenner SK, Gupta S, O’Gara BP, Krajewski ML, Charytan DM, et al. Extracorporeal membrane oxygenation in patients with severe respiratory failure from COVID-19. Intensive Care Med 2021; 47:208-21
12. Urner M, Barnett AG, Bassi GL, Brodie D, Dalton HJ, Ferguson ND, et al. Venovenous extracorporeal membrane oxygenation in patients with acute covid-19 associated respiratory failure: comparative effectiveness study. BMJ 2022; 377:e068723
13. Hajage D, Combes A, Guervilly C, Lebreton G, Mercat A, Pavot A, et al. Extracorporeal Membrane Oxygenation for Severe Acute Respiratory Distress Syndrome Associated with COVID-19: An Emulated Target Trial Analysis. Am J Respir Crit Care Med. 2022; 206(3):281-94.
